# Anabolic-androgenic steroids are linked to depression and anxiety in male bodybuilders: the hidden psychogenic side of anabolic androgenic steroids

**DOI:** 10.1080/07853890.2024.2337717

**Published:** 2024-04-08

**Authors:** Baris Karagun, Selin Altug

**Affiliations:** a Division of Endocrinology and Metabolism, Toros State Hospital, Mersin, Turkey; b Division of Psychiatry, Toros State Hospital, Mersin, Turkey

**Keywords:** Anabolic androgenic steroids, anxiety, bodybuilder, depression

## Abstract

**Background:**

The prevalence of anabolic-androgenic steroids (AAS) use is on the rise among athletes and bodybuilders worldwide. In addition to the well-documented adverse effects on hepatic, renal, and reproductive functions, there is an increasing recognition of psychiatric complications associated with AAS use. This study aimed to investigate psychiatric morbidity among male bodybuilders who are AAS users.

**Methods:**

In this cross-sectional study, 25 male bodybuilders using AAS (mean age 31.2 ± 8.9 years) were compared with a control group of 25 healthy male bodybuilders matched in age (31.3 ± 5.5 years). The demographic, hormonal, and biochemical parameters of the participants were recorded. The impact of AAS use on psychiatric morbidity was assessed using the Beck Anxiety Inventory (BAI) and Beck Depression Inventory (BDI) in both groups.

**Results:**

The BDI and BAI scores were significantly higher in male bodybuilders using anabolic-androgenic steroids (*p* < 0.0001). While the control group showed no instances of anxiety, seven individuals in the AAS user group reported mild anxiety. No participants in the control group exhibited depression, whereas seven AAS users displayed depressive symptoms (4 mild, 3 moderate). Correlations were observed between lactate dehydrogenase (LDH) levels and BAI scores, creatinine levels and both BAI and BDI scores, as well as between estradiol levels and BDI.

**Conclusion:**

The study concluded that AAS use among male bodybuilders is associated with elevated levels of depression and anxiety. Our findings suggest a potential correlation between anxiety and depression levels and the levels of creatinine, LDH, and estradiol in AAS users.

## Introduction

Anabolic-androgenic steroids (AAS) are synthetic derivatives of testosterone [1]. These substances are a diverse collection of chemicals that include endogenously produced androgens like testosterone along with their synthetically created analogues [2]. The utilization of AAS encompasses a limited number of medicinal applications. The most important therapeutic indication is endocrine dysfunction of the testes and of the hypothalamus-pituitary-gonadal axis [1]. However, performance athletes also use them illegally to increase muscle strength and promote a more muscular physical appearance [3]. Long-term use of AAS is associated with several detrimental effects on the liver as well as cardiovascular, reproductive, musculoskeletal, endocrine, and renal functions [4]. Various studies have demonstrated the psychiatric adverse effects of anabolic steroids [3,5–7]. AAS use was associated with irritability, mood swings, violent feelings, anger, hostility, distractibility, forgetfulness, and confusion [6]. Some of the underlying mechanisms of psychiatric abnormalities seen with these drugs are impaired neurotransmitter metabolism, increased oxidative stress, and inhibition of progesterone metabolism in the cortex [4,8,9]. AAS have been demonstrated to elicit pharmacological impacts on the central nervous system (CNS) through two distinct mechanisms. The first mechanism is believed to entail a direct modification of intracellular receptors, while the second is thought to induce indirect effects on the neurotransmitter receptor’s binding site or by triggering the release of neuropeptides [10,11].

Conducting prospective studies to investigate the impact of AAS on bodybuilders poses challenges due to ethical concerns associated with testing a potentially hazardous substance, and the majority of studies conducted thus far have assessed dosages that are lower than the amounts commonly reported by users [12,13]. The literature has provided limited discussion on the presence of negative psychological symptoms linked to supraphysiological doses of AAS. The objective of our study was to examine anxiety and depression levels in male bodybuilders who use anabolic-androgenic steroids (AAS) at supraphysiological doses, with a focus on identifying the related biochemical and hormonal parameters.

## Methods

This cross-sectional study was conducted at the Toros State Hospital between October and December 2022. Twenty-five male bodybuilders who were AAS users participated in the study, and twenty-five age- and sex-matched individuals served as the control group. AAS users were questioned about the anabolic steroid compounds they used and the duration of exposure. All AAS users reported the use of more than one compound. The most commonly used AASs were oxandrolone, clenbuterol, trenebolon, boldenone, testosterone propionate, testosterone enanthate, and testosterone acetate. The participants in this study were bodybuilders who had utilized anabolic steroids for a duration of one year or longer and had been actively involved in bodybuilding for a minimum of one year. In this study, although the duration may differ from one individual to another, the average cycle for bodybuilders is 10.5 weeks (range, 7–13 weeks), followed by a withdrawal phase with a mean duration of 8 weeks (range, 6–14 weeks). The control group consisted of healthy volunteers who engaged in bodybuilding for a minimum of one year without the use of any steroid derivatives. The medical records of all individuals in both the control and study groups were thoroughly examined using the Turkey National Personal Health Record System (the e-Nabız). None of the participants had a history of psychiatric diagnosis or had utilized psychiatric medications. Individuals with such characteristics were excluded from the study. We excluded participants who were not willing to participate in the study and who were under the age of 18 and over 50. All participants provided written informed consent, which included details about the study’s purpose and justification.

The hemogram and blood biochemistry parameters were assessed in both AAS (anabolic-androgenic steroid) users and controls using samples collected at 8 am The following parameters were included in the evaluation: fasting plasma glucose (FPG), serum creatinine, urea, triglycerides, total cholesterol (Total-C), low-density lipoprotein-cholesterol (LDL-C), high-density lipoprotein-cholesterol (HDL-C), alanine aminotransferase (ALT), aspartate aminotransferase (AST), gamma-glutamyl transferase (GGT), alkaline phosphatase (ALP), thyroid-stimulating hormone (TSH), free thyroxine (FT4), follicle-stimulating hormone (FSH), luteinizing hormone (LH), lactate dehydrogenase (LDH), estradiol, prolactin, cortisol, and total testosterone (TT). A random spot urine sample was collected on the test day, and the urine protein-creatinine ratio (Up/Uc) was calculated. By dividing the weight (in kilograms) by the square of the height (in meters), the body mass index (BMI) was calculated.

### Identification of psychiatric effects

The Beck Depression Inventory (BDI) is a 21-item self-report rating inventory that measures characteristic attitudes and symptoms of depression [14]. The Beck Anxiety Inventory (BAI) is a widely used 21-item self-report inventory used to assess anxiety levels [15]. BDI and BAI were used to evaluate depression and anxiety in the AAS user and control groups. The study investigators filled out the surveys using the face-to-face interview technique with the study participants. Each participant in the study was individually interviewed, and the survey questions were posed directly to them. Each participant provided their own responses to the survey questions.

### Statistical analysis

The data were analyzed using the SPSS (Statistical Package for Social Sciences) 16.0 software. In the descriptive analyses, frequency data were presented as both the number (n) and percentage (%), while numerical data were expressed as the arithmetic mean ± standard deviation (interquartile range (IQR)). Categorical data were compared using Fisher’s exact chi-square test.

To assess the normal distribution of numerical data, the Shapiro-Wilk test was employed. The distribution of normally distributed numerical data in two independent groups was examined using the Independent Samples *T* test. For non-normally distributed numerical data in independent groups, the Mann-Whitney *U* test was utilized.

The relationship between two non-normally distributed numerical variables was explored through Spearman correlation analysis. Correlation relationships were categorized as follows: low correlation if *r* = 0.05–0.30, low-moderate correlation if *r* = 0.30–0.40, moderate correlation if *r* = 0.40–0.60, good correlation if *r* = 0.60–0.70, very good correlation if *r* = 0.70–0.75, and excellent correlation if *r* = 0.75v1.00.

The diagnostic performance of parameters in predicting the presence of moderate-to-severe anxiety and moderate-to-severe depression was assessed using ROC (Receiver Operating Characteristics) curve analysis. The ROC curve results included sensitivity, specificity, and the area under the curve (AUC), provided with confidence intervals (CI).

Additionally, linear regression analysis was performed using variables to predict the factors influencing both BDI and BAI scores. The designated statistical significance threshold for all tests was established at *p* < 0.05.

## Results

The mean age for the control group was 31.3 ± 5.5 years, while for the AAS user group, it was 31.2 ± 8.9 years (*p* = 0.939). The mean length of steroid exposure in participants using AAS was found to be 2.44 ± 2.04 (1.0–3.0) years. The distributions of the hematological, biochemical, and hormonal parameters among the study participants are listed in Table 1. The mean BMI value of the control group was significantly lower compared to that of the AAS user group (*p* = 0.003).

**Table 1.** Distribution of demographic, hematological, biochemical, and hormonal parameters in anabolic steroid user and non-user groups.

Variables	Control Group (*n* = 25)	AAS users (*n* = 25)	*p*
Age (years)	31.3 ± 5.5 (27.0–35.5)	31.1 ± 8.8 (23.0–39.0)	0.683*
BMI (kg/ m^2)^	24.2 ± 1.9 (23.0–25.9)	26.5 ± 3.2 (24.8–28.8)	0.003**
Hemoglobin (g/dL)	15.0 ± 0.8 (14.4–15.7)	15.5 ± 0.9 (14.9–16.1)	0.077**
Wbc (10^3^/uL)	6.6 ± 1.2 (5.8–7.8)	7.7 ± 1.9 (6.4–8.7)	0.020**
Platelet (10^3^/uL)	222.5 ± 57.5 (169.0–270.0)	262.9 ± 68.1 (217.0–318.0)	0.028**
ALT (IU/L)	23.8 ± 9.4 (16.5–30.0)	56.9 ± 32.3 (34.5–78.0)	<0.001*
AST (IU/L)	23.2 ± 6.4 (19.0–26.5)	44.9 ± 23.4 (27.0–59.0)	<0.001*
LDH (IU/L)	161.2 ± 22.7 (143.5–178)	199.5 ± 50.6 (165.0–227)	0.002**
GGT (U/L)	25.4 ± 12.9 (15.0–31.5)	21.6 ± 8.9 (14.5–27.0)	0.346*
ALP (U/L)	56.0 ± 18.1 (40.5–70.5)	70.4 ± 20.0 (60.0–76.5)	0.011**
CK (U/L)	161.2 ± 88.9 (92.5–245.5)	730.9 ± 475.0 (278.5–1166.5)	<0.001*
LDL-C (mg/dL)	126.8 ± 33.7 (104.5–146)	111.1 ± 35.1 (84.5–133)	0.113**
HDL-C (mg/dL)	47.4 ± 8.9 (43–50.5)	44.6 ± 11.3 (39.0–46.5)	0.075*
Triglycerides (mg/dL)	129.9 ± 60.3 (79.5–179.0)	95.2 ± 36.4 (67.0–127.5)	0.018**
Total C (mg/dL)	201.6 ± 36.4 (181.5–215.0)	172.4 ± 36.5 (149.0–198.0)	0.007**
FPG (mg/dL)	88.4 ± 6.9 (84.0–92.5)	91.4 ± 13.8 (81.0–97.0)	0.340**
Sodium (mEq/L)	138.6 ± 1.6 (138.0–139.0)	139.0 ± 1.6 (138.0–140.0)	0.449*
Potassium (mEq/L)	4.3 ± 0.3 (4.0–4.6)	4.5 ± 0.3 (4.2–4.8)	0.055**
Creatinine (mg/dL)	0.9 ± 0.1 (0.8–0.9)	1.0 ± 0.1 (0.9–1.2)	0.001**
Urea (mg/dL)	32.9 ± 8.1 (27.0–37.5)	38.5 ± 8.8 (31.4–43.0)	0.009*
TSH (mIU/L)	1.9 ± 0.9 (1.2–2.1)	2.5 ± 1.2 (1.6–3.8)	0.079*
FT4 (ng/dL)	1.0 ± 0.1 (0.9–1.2)	1.1 ± 0.1 (1.1–1.2)	0.009*
FSH (mIU/mL)	4.2 ± 1.5 (3.3–4.9)	2.0 ± 1.9 (0.4–3.7)	<0.001*
LH (mIU/mL)	4.6 ± 1.4 (3.5–5.9)	2.0 ± 2.1 (0.1–4.0)	<0.001*
Testosterone (ng/mL)	461.6 ± 130.5 (374.0–576.5)	841.5 ± 458.6 (534.0–1500.0)	<0.001*
Cortisol (ug/dL)	18.3 ± 3.7 (15.0–20.5)	18.9 ± 3.4 (16.4–22.0)	0.587**
Eastridol (pg/mL)	29.1 ± 8.6 (23.2–34.2)	33.6 ± 12.9 (25.7–42.2)	0.154*
Prolactin (ng/mL)	8.6 ± 4.2 (6.0–9.7)	7.1 ± 4.1 (5.2–8.9)	0.190*
Up/Uc (mg/g)	49.4 ± 16.7 (37.6–60.2)	65.2 ± 28.0 (48.4–73.4)	0.009*

Mean **±** Standard Deviation (IQR).

*: Mann Whitney *U* Test.

**: Independent Samples *T* Test.

AAS: anabolic-androgenic steroids; ALP: alkaline phosphatase; ALT: alanine aminotransferase; AST: aspartate aminotransferase; BMI: Body Mass Index; FPG: fasting plasma glucose; GGT: gamma-glutamyl transferase; HDL-C: high-density lipoprotein-cholesterol; LDH: lactate dehydrogenase; LDL-C: low-density lipoprotein-cholesterol; PLT: platelets; Total C: total cholesterol; WBC: white blood cells. FSH: follicle stimulating hormone; FT4: free thyroxine; LH: luteinizing hormone; TT: total testosterone; TSH: thyroid stimulating hormone; Up/Uc: urine protein-to-creatinine ratio.

Bold *p* values indicate statistical significance.

Both BDI and BAI scores were significantly higher in the AAS user group than the control group (*p* = < 0.001). The control group did not exhibit any patients with anxiety, whereas the AAS user group displayed a prevalence of mild anxiety in 28% of its members. Mild depressive symptoms were noted in four participants, with three displaying symptoms of moderate severity in the AAS user group. In contrast, no individuals exhibiting depressive symptoms were detected in the control group (Table 2 and Table 3).

**Table 2.** Depression and anxiety scores of the anabolic-androgenic steroid users and the control groups.

	AAS^a^ user group median (min–max)	Control group median (min–max)	*p* value
Beck Anxiety Inventory score	5.8 ± 3.7 (2.5–9.0)	1.2 ± 1.2 (0.0–2.0)	<0.001*
Beck Depression Inventory score	6.4 ± 6.7 (1.5–11.0)	0.8 ± 1.4 (0.0–1.0)	<0.001*

Mean **±** Standard Deviation (IQR).

^a^AAS, anabolic-androgenic steroids.

*: Mann Whitney *U* Test.

**Table 3.** Distribution of depression and anxiety scores of the anabolic-androgenic steroid users and the control.

	AAS^a^ user group		Control group		*p* value
	*n*	%	*n*	%	
Beck Anxiety Inventory scores					
Normal (<8)	18	72	25	100	0.010*
Mild anxiety symptoms (8–15)	7	28	0	0	
Moderate anxiety symptoms (16–25)	0	0	0	0	
Severe anxiety symptoms (26–63)	0	0	0	0	
Beck Depression Inventory scores					
Normal (<10)	18	72	25	100	0.012*
Mild depressive symptoms (10–16)	4	16	0	0	
Moderate depressive symptoms (17–29)	3	12	0	0	
Severe depressive symptoms (30–63)	0	0	0	0	

^a^AAS, anabolic-androgenic steroids.

* Fisher Exact Testing.

**Figure 1.**
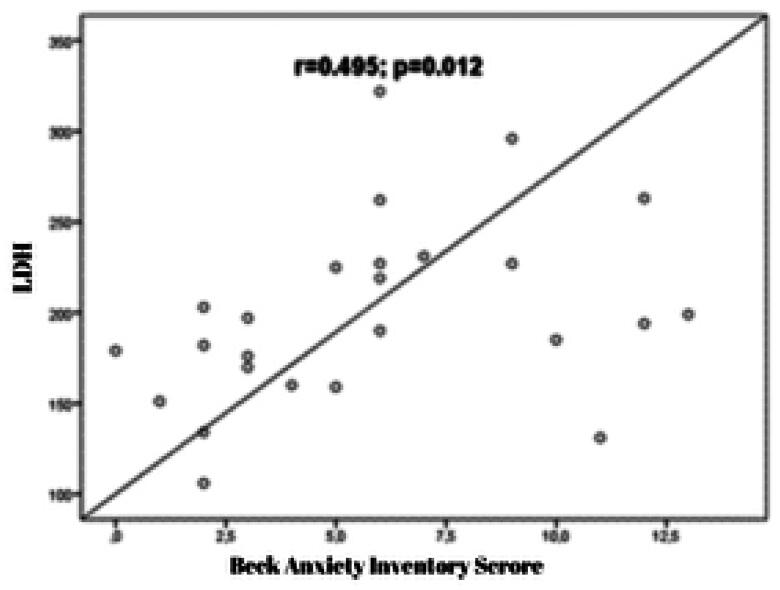
The correlation graph between LDH (lactate dehydrogenase) and BAI (Beck Anxiety Inventory) scores.

**Figure 2.**
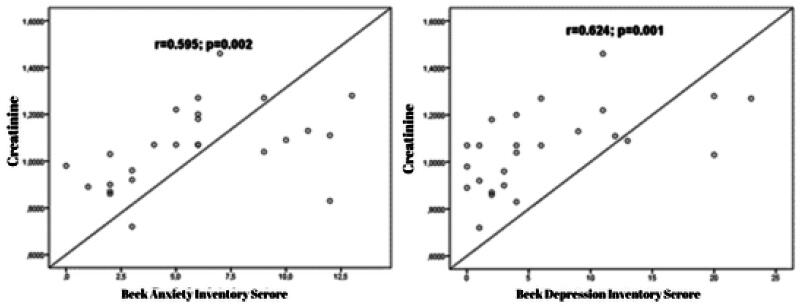
The correlation graph between BAI (Beck Anxiety Inventory) scores, BDI (Beck Depression Inventory) scores, and creatinine.

**Figure 3.**
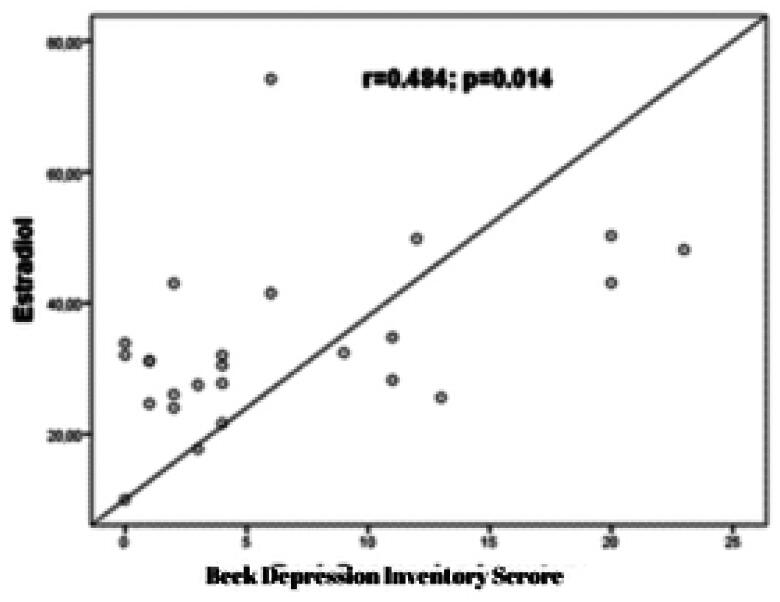
The correlation graph between estradiol and BDI (Beck Depression Inventory) scores.

The study examined the relationship between laboratory parameters and BAI and BDI scores. The results showed that a moderately significant positive correlation was observed between LDH and BAI scores (*r* = 0.495; *p* = 0.012) (Figure 1). Additionally, a moderately significant positive correlation was observed between creatinine and BAI scores (*r* = 0.595; *p* = 0.002). A significant positive correlation was found between creatinine and BDI scores (*r* = 0.624; *p* = 0.001) (Figure 2). Furthermore, a moderately significant positive correlation was observed between estradiol level and BDI scores (*r* = 0.484; *p* = 0.012) (Figure 3).

**Figure 4.**
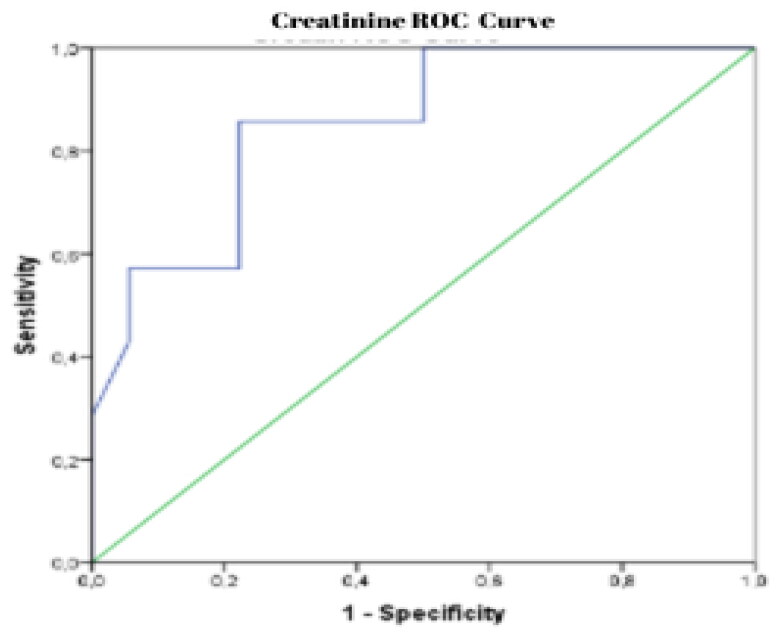
Receiver operating characteristic (ROC) curve of creatinine (sensitivity: 85.7% and specificity: 87.8%, AUC: 0.853).

ROC curve analysis was conducted to determine the cut-off point of creatinine in predicting moderate-to-severe depression in anabolic steroid users. The analysis showed that creatinine values of 1.08 and above could predict the diagnosis of moderate-severe depression with 85.7% sensitivity and 87.8% specificity (AUC: 0.853 (CI: 0.693–0.999); *p* = 0.007) (Figure 4).

A regression analysis was conducted to predict anxiety. The results indicated that there was a statistically significant increase in BAI scores associated with an increase in creatinine (*p* < 0.001), as well as an increase in BAI scores associated with an increase in LDH (*p* = 0.039) (Table 4).

**Table 4.** Regression analysis results for beck anxiety Inventory score.

	B (%95 CI)	Beta	*t*	*p*	Zero-order	Partial
Constant				<0.001		
Creatinine	10.319 (4.940–15.699)	0.470	3.859	<0.001	0.544	0.491
LDH	6.375 (1.345–12.404)	0.259	2.127	0.039	0.393	0.296

B: Unstandardized Coefficient, Beta: Standardized Coefficient.

LDH: lactate dehydrogenase.

A regression analysis was conducted to estimate depression. The results showed that an increase in creatinine was associated with a significant increase in BDI scores (*p* < 0.001), while an increase in estradiol was also found to be associated with a significant increase in BDI scores (*p* = 0.028) (Table 5).

**Table 5.** Regression analysis results for beck depression Inventory score.

	B (%95 CI)	Beta	*t*	*p*	Zero-order	Partial
Constant				<0.001		
Creatinine	16.973 (8.728–25.219)	0.497	4.141	<0.001	0.601	0.517
Eastridol	0.137 (0.015-0.258)	0.272	2.266	0.028	0.463	0.314

B: Unstandardized Coefficient, Beta: Standardized Coefficient.

## Discussion

The purpose of this study was to investigate the psychiatric adverse effects of supraphysiological doses of anabolic steroid use on male bodybuilders. The most notable findings of this study indicate that the use of AAS was correlated with heightened levels of depression and anxiety. Depression and anxiety symptoms were found to be more prevalent in the AAS user group compared to the control group. Our study revealed significant correlations between BDI and BAI scores and the levels of creatine, LDH, and estradiol in AAS users. This finding represents a valuable contribution to the evolving understanding of AAS-related mental health outcomes, as such associations have been sparsely investigated in previous research.

AAS are chemicals used by athletes for performance-enhancing purposes but may have many side effects [4,16]. AAS has the potential to upset the natural hormonal equilibrium in the body, resulting in mood swings, irritability, and a possible contribution to depression and anxiety [5]. They could potentially affect neurotransmitter systems in the brain, notably serotonin and dopamine, which have a role in regulating mood, and these disruptions are thought to contribute to the symptoms of anxiety and depression [11]. On the other hand, AAS use can also trigger depression and anxiety during the withdrawal period [5]. Individuals who use AAS are found to be at a notable risk of experiencing major depressive episodes within the initial months after discontinuing the use of AAS [17]. AAS also has other psychosocial effects. The use of anabolic steroids is occasionally linked with concerns over body image and a wish to attain a specific physique, and psychological factors associated with body image, self-esteem, and societal expectations have the potential to contribute to mental health problems. [18–20]. Another concern that impacts the mental well-being of AAS users is simultaneous substance misuse. A marked increase in the consumption of cannabis, cocaine, and alcohol was noted in individuals who used AAS as opposed to those who did not use AAS. [21]. In this study, we used the BDI and BAI to detect depression and anxiety, respectively, in participants. We found that, consistent with prior research, the AAS user group displayed mild to moderate depressive symptoms. Similarly, mild anxiety was detected in the AAS user group.

AAS were shown to impair the hypothalamic-pituitary-gonadal-axis as well [22]. The effects of AAS on the reproductive system are well known, and the majority of AAS users have developed hypogonadism, with consistently low blood levels of testosterone and gonadotropin even after AAS withdrawal [23]. It was also reported that lower endogenous testosterone levels were associated with depression and anxiety [24]. In our study, the mean gonadotropin levels of AAS users were significantly lower, and testosterone levels were significantly higher. The conventional methods for detecting testosterone, however, cannot discriminate between testosterone produced endogenously and that taken exogenously unless radioimmunoassay and tandem mass spectrometry methods are used [25]. In the present study, it has been observed that the levels of testosterone are elevated in our study group as compared to the control group. This disparity can be attributed to the administration of exogenous testosterone and exogenous testosterone esters. If the endogenous testosterone levels of our patients were measured, it is probable that lower levels of endogenous testosterone would have been observed in the group of individuals using anabolic-androgenic steroids (AAS). Therefore, we hypothesize that the observed decrease in endogenous testosterone levels, as compared to the control group, resulted in an increase in the occurrence of symptoms related to depression and anxiety.

Lactate dehydrogenase (LDH) is a crucial enzyme involved in the process of glycolysis [26]. In this study, a correlation was identified between LDH levels and anxiety levels in the group using AAS. LDH levels have been established as potentially related to major depressive disorder and suicidal behavior [27]; however, the connection between LDH and anxiety remains incompletely understood. To the best of our knowledge, this is the initial study examining the association between anxiety levels and LDH levels. In athletes who use AAS, elevated LDH levels occur due to liver damage resulting from the intake of supraphysiological doses [28]. The findings from the correlation analysis revealed a positive correlation between serum LDH levels and anxiety levels in the AAS user group.

The AAS user group exhibited elevated levels of creatinine and creatinine kinase, both recognized indicators of organ damage. Furthermore, there was a noteworthy positive correlation between creatinine levels and the levels of anxiety and depression. This suggests a potential association between AAS use, organ damage, and adverse psychological effects. Exogenous creatinine comes from eating and breaking down animal products. Endogenous creatinine is produced by the muscles’ metabolism. Blood creatinine is made up of both types of creatinine [29]. Its connection to mental health conditions, including depression, is not fully understood. Although creatinine levels are not typically considered a direct factor in mental health or depression, abnormalities in kidney function that can affect creatinine levels may be associated with mental well-being, and it has been suggested that chronic kidney disease may increase the risk of depression, although this relationship is likely to be multifactorial and involve factors beyond creatinine levels [30–32].

The correlation between creatinine and LDH, biochemical parameters indicative of muscle and liver damage resulting from high-dose AAS use, observed in our study suggests that higher doses of AAS may contribute to a predisposition to elevated levels of anxiety and depression.

One of the other findings of our study was that there was a positive correlation between estradiol and depression levels. The misuse of supraphysiological doses of androgens is linked to various androgen-related side effects, including consequences such as acne, gynecomastia, and baldness, and these side effects are primarily attributed to elevated levels of estradiol [33]. The increased frequency of gynecomastia in patients using AAS often necessitates surgical operation. In the study conducted by Vojvodic et al. it was found that 11% of the patients with gynecomastia used AAS, and the patients who needed reconstructive surgery were statistically significantly more likely to be athletes using AAS [34]. These consequences, attributed to the use of AAS and its impact on physical appearance, may elucidate the observed elevated levels of depression that were associated with the estradiol levels of participants in our study.

Some limitations of the present study are worthy of mention. First, this is a cross-sectional study and does not necessarily reflect the causal relationship between AAS use and observed effects. Secondly, within our study, there was variability in the utilization of AAS compounds among AAS users. Given the study’s design, the reported dosage of AAS was reliant on participants’ self-reports. It is plausible that participants disclosed lower doses than their actual usage, possibly influenced by an awareness of the prohibition associated with substance use. Furthermore, variations were observed in both the duration and frequency of AAS use among the participants. Thirdly, AAS users are a hard-to-reach group owing to several factors, including fear of litigation because of their use of illicit substances, and so the number of participants in our study was relatively low. It is important to mention that our study, being cross-sectional in nature, did not permit a baseline psychological assessment due to its design. Nevertheless, all participants underwent a thorough assessment. Crucially, none of the participants had a documented history of any psychiatric diagnosis or prior use of psychiatric medications, establishing a distinct and clear foundation for our investigation.

The strength of our study lies in its distinctive approach compared to existing studies on AAS. While the majority of research in this field relies on randomized controlled trials involving lower doses of AAS than those commonly used by athletes in real-world scenarios, our study specifically focused on individuals actively using supraphysiological doses of AAS. This unique angle allows us to explore the effects and consequences associated with supraphysiological AAS dosages to which athletes are exposed in real life, providing valuable insights that may be overlooked in studies with lower doses. Moreover, our study contributes to the existing literature by addressing a gap in research—the scarcity of cross-sectional studies specifically examining individuals using supraphysiological doses of AAS. By filling this gap, we enhance the comprehensiveness of our understanding of AAS effects and risks, shedding light on a population that has been less explored in scientific inquiry.

In conclusion, the findings of our study indicate a notable rise in the occurrence of depression and anxiety among bodybuilders who use AAS in comparison to bodybuilders who do not use these substances. The identified correlations between elevated creatinine, LDH, and estradiol levels and increased BAI and BDI scores, a key secondary finding of our study, underscore the need for vigilant monitoring of these biochemical markers in AAS users. In order to ascertain the impact of dosage and duration of AAS use on psychiatric morbidity, it is imperative to conduct longitudinal research.

## Data Availability

The data that support the findings of this study are available from the corresponding author, [B.K.], upon reasonable request.
